# Supplementary figures and images for: MEG3 Expression Indicates Lymph Node Metastasis and Presence of Cancer-Associated Fibroblasts in Papillary Thyroid Cancer
Source: Cells. 2022 Oct 10;11(19):3181. doi: 10.3390/cells11193181 (PMC9562881; doi:10.3390/cells11193181)

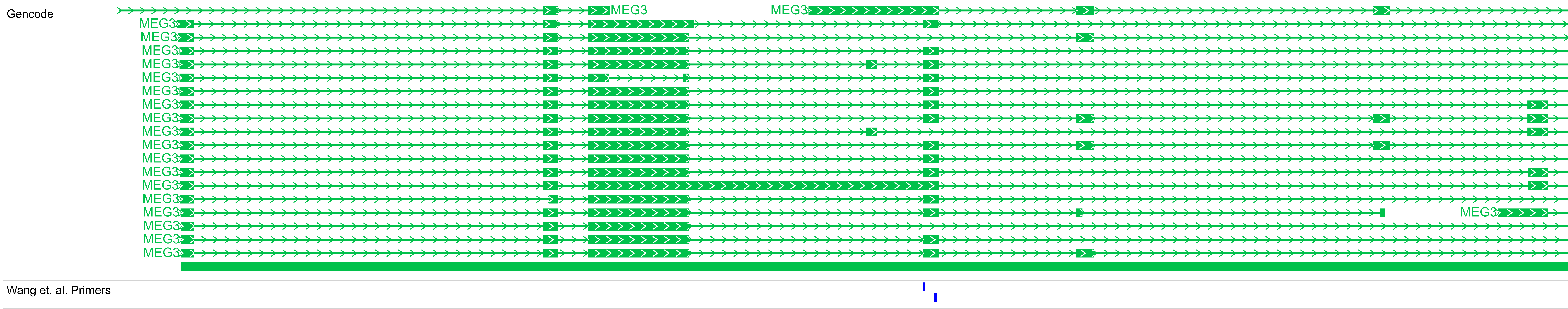

Supplement: Supplementary file 1 [file cells-11-03181-s001.zip › Supplentary Figure S1.png]
